# Epigenomics and Ayurgenomics: Molecular Analysis of Ayurvedic Prakriti in Personalized Healthcare

**DOI:** 10.7759/cureus.113434

**Published:** 2026-07-27

**Authors:** Amit R Nampalliwar, Swati N Khandale, Pooja Singh, Kavita Pradhan, Heera Chand Patel, Chandreshwar Prasad Sinha

**Affiliations:** 1 Department of Roga Nidan and Vikriti Vigyan (Diagnostic Procedures and Pathology), Government Ayurved College and Hospital, Bilaspur, IND; 2 Department of Kriya Sharira (Physiology), Institute of Teaching and Research in Ayurveda (ITRA), Jamnagar, IND; 3 Department Roga Nidan Evum Vikriti Vigyan (Diagnostics and Pathology), R.A. Podar Ayurved Medical College, Mumbai, Maharashtra University of Health Sciences, Nashik, IND; 4 Department of Rachana Sharir (Anatomy), Government Ayurved College and Hospital, Bilaspur, IND and Pandit Deendayal Upadhyay Memorial Health Sciences and Ayush University of Chhattisgarh, Raipur, IND, Bilaspur, IND; 5 Department of Kayachikitsa (Internal Medicine), Government Ayurved College and Hospital, Bilaspur, IND and Pandit Deendayal Upadhyay Memorial Health Sciences and Ayush University of Chhattisgarh, Raipur, IND, Bilaspur, IND; 6 Department of Kayachikitsa (Internal Medicine), Shri Narayan Prasad Awasthi Government Ayurved College, Raipur, IND and Pandit Deendayal Upadhyay Memorial Health Sciences and Ayush University of Chhattisgarh, Raipur, IND, Raipur, IND

**Keywords:** ayurgenomics, epigenomics, personalized healthcare, prakriti, precision medicine

## Abstract

Personalized healthcare increasingly requires models that explain individual variation in disease risk, prevention, and treatment response. Ayurvedic Prakriti offers a constitution-based framework for classifying human variability through Vata, Pitta, and Kapha phenotypes, while Ayurgenomics attempts to connect these traditional classifications with molecular biology. Despite growing interest, the biological relationship between Prakriti, epigenomic regulation, and precision healthcare remains insufficiently established, particularly because available evidence is limited by small cohorts, heterogeneous assessment tools, and incomplete biomarker validation.

This review examines the molecular relevance of Prakriti by analyzing evidence from genomics, epigenomics, transcriptomics, proteomics, metabolomics, pharmacogenomics, and systems biology. Literature was evaluated thematically to identify constitution-specific molecular signatures, disease susceptibility patterns, lifestyle-related epigenetic influences, and translational applications in individualized healthcare. Current findings suggest that Prakriti types may be associated with distinct biological profiles involving metabolism, inflammation, immune regulation, oxidative stress, stress adaptation, and drug response. Epigenomic mechanisms may further explain how diet, lifestyle, environment, aging, and stress modify gene expression within constitution-specific contexts. Integrating Ayurgenomics with multi-omics platforms may support preventive screening, personalized nutrition, lifestyle planning, and precision therapeutics. Stronger validation through standardized tools, longitudinal studies, and clinical trials remains essential.

## Introduction and background

Personalized healthcare has gradually moved the biomedical research paradigm from a disease-centric perspective to an individual-centric one, advocating the importance of taking into account personalized genetic/molecular, environmental, behavioral, and physiological variation among individuals [1]. Traditional health care systems, which have traditionally been based on the individual, particularly Ayurveda, where susceptibility to disease, preventive therapy, diet, and therapy are all defined in terms of Prakriti [2], are being rethought and reviewed in the light of this shift. Prakriti refers to the psycho-physiological makeup of an individual, which is basically determined based on the predominance of Vata, Pitta, and Kapha doshas either singly or in combination [3]. The Prakriti is a constitutional type that lasts throughout life and is present at birth and influences how the body operates, how it is constructed, its temperaments, immunity, digestive power, stress response, and susceptibility to some diseases, unlike the majority of the modern diagnostic categories, which are largely disease-oriented. However, the model is relevant in the context of personalized medicine today as it attempts to stratify the population before disease and not after symptom production [4].

Ayurgenomics is now an interdisciplinary research area to understand if there are measurable genomic, transcriptomic, proteomic, metabolomic, and physiological correlates for the Ayurvedic Prakriti classifications [5]. The main concept of Ayurgenomics is that the traditional constitution types could be a reflection of a potential biological diversity, such as differences in gene expression, immune regulation, metabolic pathways, inflammatory response, and pharmacological behavior [6]. Very few research studies have suggested that there are variations in response to hypoxia, inflammatory markers, biochemical profile, metabolic tendencies, and susceptibility to disease among the different Prakriti groups, thus making Prakriti a biologically meaningful stratification system [7]. The findings indicate that the phenotyping approach from Ayurveda could be combined with the modern precision approach of molecular phenotyping-based precision medicine, especially in areas of risk prediction, preventive measures, and personalization of treatment [8]. Therefore, Prakriti analysis and molecular biology can provide a culturally based, scientifically verifiable approach towards understanding inter-individual variability in health and disease [9].

Epigenomics regulates gene expression without altering the DNA sequence through mechanisms such as DNA methylation, histone modification, chromatin remodeling, and non-coding RNA regulation. These processes are influenced by diet, lifestyle, stress, aging, environmental exposures, and disease states [10]. This aligns closely with Ayurveda, where individualized recommendations on diet, daily routines, sleep, stress management, and seasonal adaptation may influence epigenetic regulation and shape biological responses across the lifespan. Interactions between inherited constitution, epigenetic mechanisms, and environmental factors contribute to individual differences in disease susceptibility, therapeutic response, and resilience [11].

Ayurgenomics integrates these concepts by viewing Prakriti as a systems-level phenotype reflecting genetic predisposition, epigenetic regulation, metabolism, and environmental responsiveness. Pitta individuals exhibit molecular signatures related to metabolism, inflammation, and oxidative stress, whereas Vata traits may correspond to neuroendocrine regulation, circadian rhythm, and stress-responsive epigenetic pathways. Kapha characteristics are associated with lipid metabolism, insulin signaling, adipogenesis, and inflammatory regulation [12].

This framework supports precision medicine by enabling individualized risk prediction, preventive strategies, nutrition planning, lifestyle modification, and therapeutic decision-making. Incorporating epigenomic markers with Prakriti assessment may shift healthcare from static genetic prediction toward dynamic, preventive, and personalized disease management, particularly for chronic cardiometabolic and inflammatory disorders [13].

The goal of this review is to explore the molecular link between Ayurvedic Prakriti, epigenomic regulation, and Ayurgenomic variation in the field of personalized healthcare. It also aims to assess the potential of Prakriti-based molecular profiling to assist in the prediction of disease risk, prevention, and personalized treatment.

## Review

Methodology

The molecular relevance of Ayurvedic Prakriti in relation to epigenomics, Ayurgenomics, and personalized healthcare was explored in this narrative review. Articles were searched in PubMed, Scopus, Web of Science, Google Scholar, ScienceDirect, SpringerLink, MDPI, and Frontiers. The keywords used were 'Ayurgenomics,' 'Prakriti,' 'Ayurveda,' 'epigenomics,' 'DNA methylation,' 'multi-omics,' 'personalized medicine,' 'precision healthcare,' 'pharmacogenomics,' 'metabolomics,' and 'lifestyle epigenetics.' Articles published between 2018 and 2026 were considered to have recent scientific relevance. Original research articles and review articles were selected if they discussed any of the following: Prakriti classification, molecular signatures, epigenetic regulation, disease susceptibility, lifestyle-related gene regulation, pharmacogenomics, multi-omics integration, and personalized healthcare applications. Articles were excluded if they did not address the review topic, were not peer-reviewed publications, were conference abstracts, editorials, theses, reports, or guidelines, or were available only through ResearchGate. The included literature was thematically reviewed, and the data were compiled into the major sections relating to Prakriti concepts, Ayurgenomics, epigenomics, disease risk, lifestyle modulation, pharmacogenomics, and translational multi-omics frameworks.

Review

Conceptual Basis of Ayurvedic Prakriti

In Ayurveda, Prakriti is a term that describes the psycho-physiological individuality of a person and explains inter-individual variations in health, susceptibility to illness, and response to treatment. The predominance of the functional principles called Vata, Pitta, and Kapha is traditionally the criterion used to determine an individual's Prakriti. Prakriti is independent of disease and is considered a constitutional trait that remains stable throughout life and is influenced by hereditary, developmental, environmental, dietary, and behavioral factors [14].

Vata individuals often exhibit variability in appetite, sleep, body weight, stress response, stress sensitivity, and neurological function. Pitta individuals tend to have a higher metabolic rate and exhibit greater heat production, inflammatory tendencies, and digestive activity. Individuals with Kapha predominance generally have greater structural stability, slower metabolism, higher endurance, and an increased tendency for tissue accumulation. These descriptions are not definitive but rather represent combinations of phenotypic characteristics that may occur individually or in combinations of two or three doshas, referred to as Dvandvaja (dual-dosha) and Tridoshaja (tri-dosha) constitutions, respectively [15].

**Figure 1:**
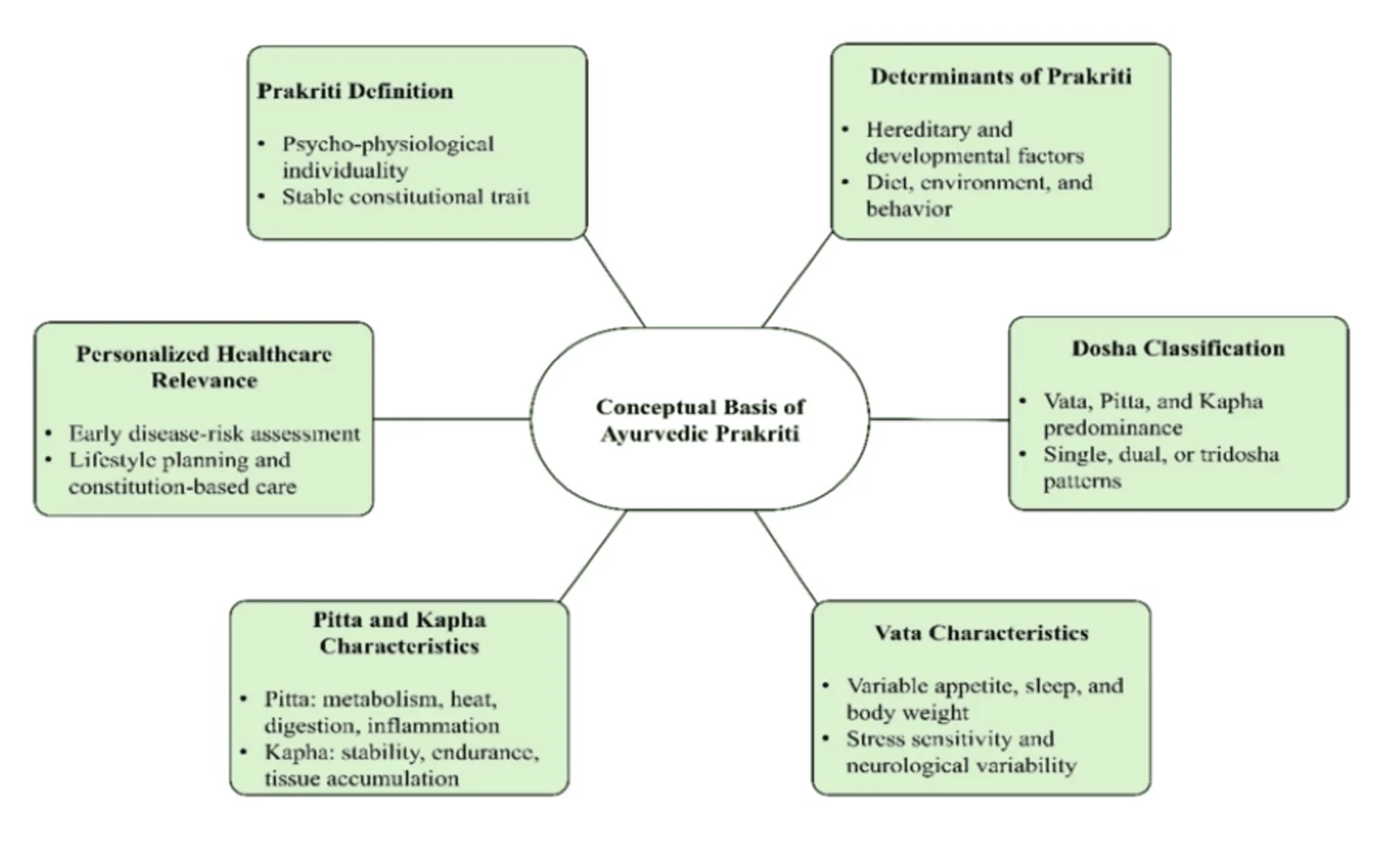
Conceptual basis of Ayurvedic Prakriti in personalized healthcare

Prakriti is a phenotype-based approach to understanding individual differences and preventing disease through personalized healthcare. It may aid in early risk assessment, preventive counseling, lifestyle planning, and therapeutic decision-making based on an individual's constitution. The relevance of Prakriti has increased with the advent of precision medicine and the recognition of disease heterogeneity in modern medicine. Prakriti may serve as an individualized health assessment system, offering a culturally based model that can be explored using molecular biology, systems medicine, and biomarker-driven research [16]. The conceptual basis of Ayurvedic Prakriti and its relevance to personalized healthcare are illustrated in Figure 1.

Integrating Ayurveda With Molecular Biology (Ayurgenomics)

The concept of Ayurgenomics is an interdisciplinary approach that seeks to correlate Ayurvedic constitutional types with molecular, genomic, transcriptomic, proteomic, metabolomic, and physiological markers. The premise of Ayurgenomics is that the biological basis of Prakriti may extend beyond traditional nomenclature and may be measurable. This can be assessed using modern molecular tools and could assist in understanding constitution-specific biological pathways relevant to disease risk, prevention, and individualized treatment through an Ayurveda-based phenotyping approach [17].

Several studies have suggested that genetic polymorphisms, gene expression levels, immune responses, metabolic traits, inflammatory markers, and other physiological characteristics may differ among Prakriti groups. These observations indicate that broad biological trends identified through traditional constitutional assessment may overlap with molecular endotypes. For example, genetic polymorphisms related to metabolism, hypoxia response, inflammation, detoxification, and oxidative stress have been investigated in relation to Prakriti groups. The findings suggest that Prakriti is not determined by a single gene but rather reflects the interaction of multiple biological systems [18].

The true strength of Ayurgenomics lies in its ability to integrate traditional Ayurvedic principles with the concept of personalized medicine in an evidence-based manner. It provides an opportunity to investigate differences in disease course, response to therapy, and life experiences among individuals exposed to similar factors [19]. Assessment of Prakriti, together with genomic and molecular profiles, could potentially be used for risk stratification, preventive screening, interpretation of genomic and molecular profiles, and planning individualized interventions. To facilitate clinical translation, however, the field requires further validation in larger cohorts, standardized assessment tools for Prakriti, reproducible molecular endpoints, and multicenter studies [20].

Epigenomics and Human Phenotypic Variation

Epigenomics is a term used to describe genome-wide mechanisms that alter gene expression without changing the DNA sequence. The key epigenetic mechanisms are DNA methylation, histone modification, chromatin remodeling, and regulation of gene expression by non-coding RNA [21]. They control gene accessibility, transcription, cell differentiation, immunity, metabolism, and adaptive responses to environmental stimuli. Epigenomic regulation, therefore, is an important mechanism for explaining how the same genotype may give rise to different phenotypes, disease susceptibilities, and physiological responses [22].

The epigenome is highly responsive to environmental and lifestyle exposures. Epigenetic patterns can change over time in response to diet, exercise, sleep patterns, emotional stress, exposure to toxins, the gut microbiome, aging, and inflammatory states [23]. These alterations may affect insulin sensitivity, lipid metabolism, immune regulation, neuroendocrine regulation, oxidative stress, and cellular aging pathways. Many of these exposures are modifiable and therefore represent suitable targets for preventive and lifestyle-based healthcare approaches that incorporate epigenomic principles [24].

**Figure 2:**
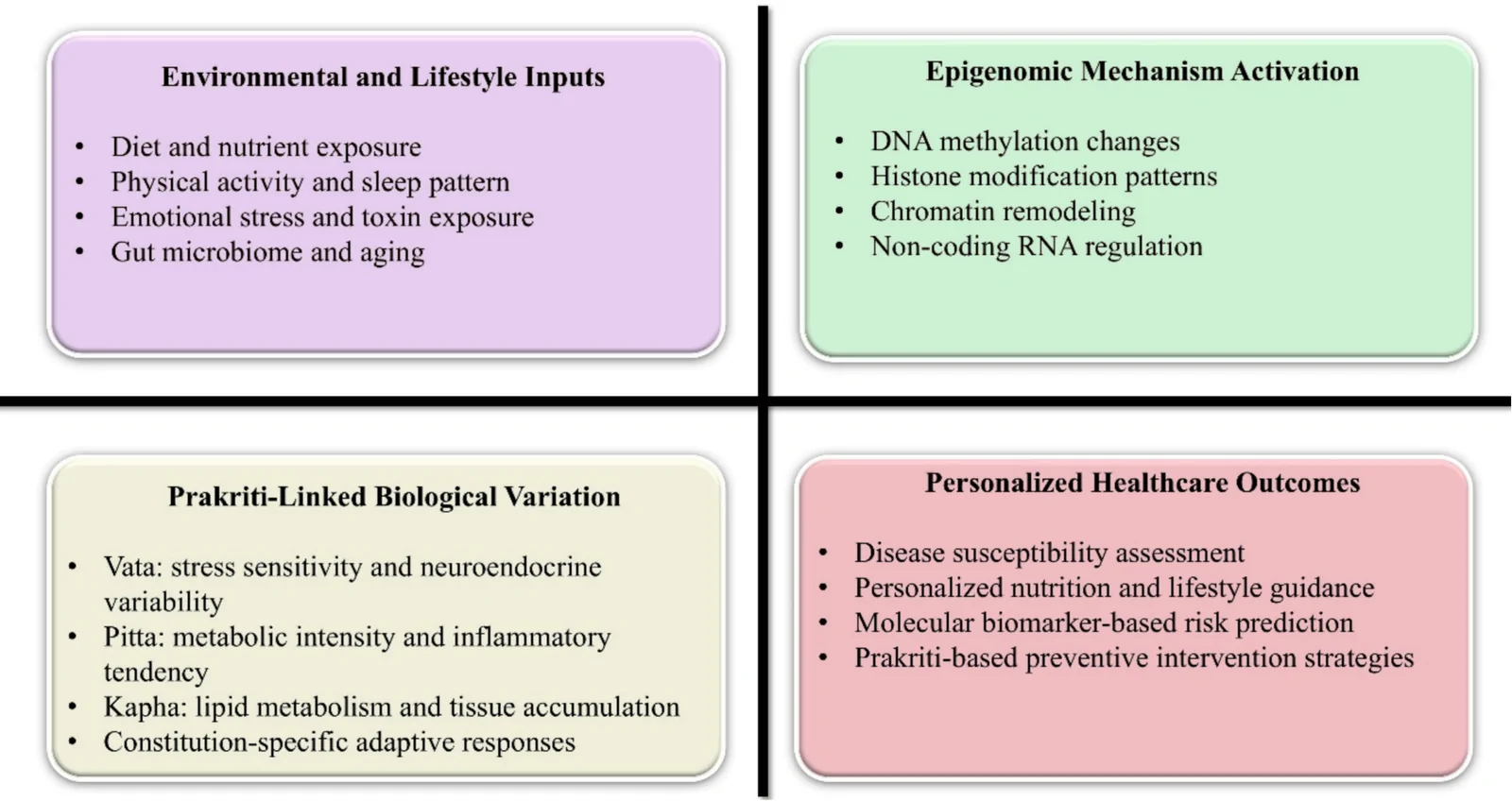
Epigenomics and Prakriti-linked healthcare outcomes

Within Ayurveda, the relevance of the epigenomic framework can be appreciated because its healthcare recommendations are individualized and based on diet, daily routine, seasonal adaptation, stress management, sleep regulation, and behavioral balance [25]. These practices may exert epigenetic effects on biological regulation; however, direct evidence linking specific Ayurvedic practices to specific epigenomic responses remains limited [26]. Epigenomics also provides a framework for understanding how environmental factors interact with an individual's Prakriti to influence disease susceptibility and adaptive responses. This concept positions epigenomics as a potential molecular bridge between the classical phenotypic classification of Ayurveda and modern precision medicine models [27]. Epigenomics-related Prakriti variation is summarized in Figure 2.

Molecular Signatures Associated With Prakriti

The molecular investigation of Prakriti aims to identify biological markers and understand their roles in health and disease, thereby helping to differentiate Prakriti types [28]. Reported molecular signatures include genetic variations, gene expression profiles, serum biochemical patterns, inflammatory markers, metabolomic profiles, and other molecular differences. These markers may reflect differences in metabolism, immunity, thermoregulation, tissue structure, stress response, and detoxification capacity among individuals with different Prakriti constitutions. Collectively, the available evidence supports the concept that Prakriti is a complex phenomenon involving multiple interconnected biological pathways.

Genetic studies have investigated associations between Prakriti and polymorphisms in genes involved in immune regulation, hypoxia adaptation, metabolism, and inflammatory signaling. Transcriptomic differences have also been reported among Prakriti groups, suggesting variation in pathway activation even among healthy individuals [29]. More recently, metabolomics and proteomics have been proposed as approaches for identifying constitution-specific biochemical states, including differences in lipid metabolism, amino acid turnover, oxidative stress markers, and inflammatory mediators [30].

Epigenetic signatures may further enhance the molecular understanding of Prakriti by providing insight into the regulatory mechanisms that link constitutional attributes with environmental responsiveness. Physiological characteristics and disease susceptibility can differ among individuals with the same genotype, potentially owing to variations in DNA methylation, histone modification, and non-coding RNA regulation. However, the molecular findings from Prakriti studies remain preliminary because of small sample sizes, inconsistent classification methods, and limited replication. Studies integrating multi-omics platforms with standardized Prakriti assessment and longitudinal follow-up will be valuable for evaluating the predictive, preventive, and therapeutic utility of these molecular signatures [31]. A summary of the molecular correlates of Prakriti is presented in Table 1.

**Table 1:** Molecular and omics-level correlates of Ayurvedic Prakriti types SNPs: single nucleotide polymorphisms; DNA: deoxyribonucleic acid; RNA: ribonucleic acid

Prakriti-related molecular domain	Key biological focus	Reported or proposed molecular relevance	Personalized healthcare implication
Genomic variation	SNPs, inherited variants, pathway-level genetic differences	May contribute to constitution-specific traits involving metabolism, immunity, inflammation, and stress response	Supports inherited risk stratification and molecular classification of Prakriti groups
Transcriptomic profiles	Gene expression differences across Prakriti types	May reflect variation in active biological pathways, including immune, metabolic, and adaptive-response pathways	Useful for identifying active molecular signatures linked to constitutional phenotypes
Epigenomic regulation	DNA methylation, histone modification, and non-coding RNA activity	May explain how diet, lifestyle, stress, and environment influence Prakriti-linked biological responses	Supports dynamic, modifiable biomarker-based personalized prevention
Proteomic markers	Functional proteins and signaling molecules	May indicate differences in inflammatory, metabolic, enzymatic, and structural protein expression	May help identify constitution-specific disease risk or treatment response profiles
Metabolomic patterns	Lipids, amino acids, energy metabolites, oxidative stress markers	May represent real-time biochemical variation among Prakriti types	Supports personalized nutrition, metabolic risk prediction, and lifestyle planning
Integrated multi-omics	Combined genomic, epigenomic, transcriptomic, proteomic, and metabolomic profiling	May provide a systems-level explanation of Prakriti as a complex biological phenotype	Enables biomarker panels and predictive models for precision healthcare

Prakriti, Disease Susceptibility, and Preventive Healthcare

In Ayurveda, Prakriti-based classification has long been used to estimate an individual's susceptibility to disease and to guide preventive healthcare measures accordingly. Different constitutional types are believed to have varying predispositions to metabolic, inflammatory, neurological, digestive, and structural disorders [29]. In contemporary research, Prakriti is increasingly viewed within the framework of precision medicine, which recognizes that disease susceptibility varies among individuals because of genetic, molecular, environmental, behavioral, and physiological factors. Therefore, if supported by systematic biomedical evidence, Prakriti could serve as a phenotypic marker for risk stratification [30,31].

Pitta-dominant individuals are thought to be more susceptible to inflammatory, acid-peptic, heat-related, and metabolic disorders, potentially through mechanisms involving oxidative stress, cytokine signaling, enzymatic activity, and energy metabolism. Vata-dominant individuals may be more prone to anxiety, sleep disturbances, neurological variability, musculoskeletal complaints, and stress-related dysregulation, possibly involving neuroendocrine and autonomic pathways [30]. Kapha-dominant individuals may have a greater tendency toward obesity, dyslipidemia, insulin resistance, congestion, and slower metabolism, suggesting possible associations with adipogenesis, lipid metabolism, and low-grade inflammation [31].

Risk stratification based on Prakriti may be valuable for preventive healthcare when supported by molecular and clinical evidence linking constitutional type to disease risk. Lifestyle modification, physical activity recommendations, dietary planning, stress management, and appropriate screening schedules may all form part of a Prakriti-based preventive strategy. This approach could be further strengthened by incorporating epigenomic markers to identify modifiable biological changes before disease onset. However, clinical implementation requires validated associations between Prakriti types and disease outcomes, standardized assessment tools, and prospective studies demonstrating that Prakriti-based interventions produce measurable improvements in health outcomes [32]. The disease tendencies associated with different Prakriti types are summarized in Table 2.

**Table 2:** Prakriti and disease susceptibility Note: Vata, Pitta, and Kapha: Ayurvedic constitutional principles; dosha: functional regulatory principle in Ayurveda.

Prakriti type	Traditional physiological tendency	Potential biomedical correlation	Possible disease susceptibility	Preventive healthcare relevance
Vata-dominant	Variability, lightness, sensitivity, irregular appetite, and sleep	Neuroendocrine variability, autonomic imbalance, stress responsiveness, and circadian dysregulation	Anxiety, sleep disturbance, musculoskeletal complaints, stress-related disorders	Routine, sleep stabilization, stress reduction, nourishment-focused lifestyle planning
Pitta-dominant	Heat, sharp digestion, metabolic intensity, and inflammatory tendency	Increased metabolic activity, oxidative stress, cytokine signaling, and inflammatory pathway activation	Acid-peptic disorders, inflammatory conditions, metabolic disturbances, and heat-related symptoms	Anti-inflammatory dietary planning, cooling lifestyle practices, and metabolic monitoring
Kapha-dominant	Stability, heaviness, slower metabolism, tissue-building tendency	Lipid metabolism variation, adipogenesis, insulin resistance, and low-grade inflammation	Obesity, dyslipidemia, metabolic syndrome, congestion-related conditions	Physical activity, a lighter diet, weight monitoring, and early cardiometabolic screening
Dual-dosha constitution	Mixed features of two dominant doshas	Combined metabolic, inflammatory, neurological, or structural tendencies	Variable disease susceptibility depending on dominant functional traits	Individualized preventive strategy based on a combined constitutional profile
Tridosha or balanced constitution	Relative equilibrium of Vata, Pitta, and Kapha	Greater physiological balance but still influenced by environment and lifestyle	Lower constitutional predisposition, but risk may emerge through lifestyle or exposure imbalance	Maintenance-focused prevention, seasonal adaptation, and routine health surveillance

Diet, Lifestyle, and Environmental Modulation of the Epigenome

Food and the food environment are important contributors to epigenetic programming and may influence disease risk through DNA methylation, histone modification, microRNA expression, oxidative balance, inflammation, and metabolic signaling. Nutrients such as polyphenols, fatty acids, vitamins, and bioactive plant compounds influence pathways involved in antioxidant activity, inflammatory regulation, and methylation processes [33]. Other factors, including physical activity, sleep quality, circadian rhythm, psychological stress, and environmental exposures, also influence epigenetic plasticity throughout the lifespan [34].

A key focus of Ayurveda is individualized dietary guidance based on constitution, daily regimen, seasonal changes, digestion, sleep habits, and mental equilibrium. These recommendations are sometimes tailored according to the Prakriti (constitution)-based approach to lifestyle medicine. Dietary and lifestyle practices for Pitta individuals may be directed toward reducing excess heat and inflammation, whereas those for Kapha individuals may aim to stimulate metabolism through lighter foods and increased activity. Generally, Vata individuals are guided toward principles of stability, nourishment, regularity, and stress reduction [35].

At the molecular level, these personalized practices could influence the epigenetic regulation of these processes through alterations in metabolic state, inflammatory burden, gut microbiota, neuroendocrine function, and oxidative stress. This provides a possible mechanism through which Ayurvedic lifestyle principles may be correlated with modern preventive medicine. However, current evidence remains largely circumstantial, and few studies have investigated whether dietary and lifestyle modifications tailored to Prakriti produce measurable changes in the epigenome. The effectiveness of constitution-guided interventions in modifying epigenetic markers and improving clinical outcomes in chronic disease prevention should be evaluated through well-designed longitudinal trials [36].

Pharmacogenomics and Personalized Therapeutics

Pharmacogenomics involves understanding the role of genetic variation in the absorption, distribution, metabolism, efficacy, and toxicity of drugs. It is an essential component of precision medicine because individuals may show significant variations in treatment response and adverse effects. In Ayurvedic medicine, treatment response is considered to vary according to multiple factors, including Prakriti, digestive capacity, tissue strength, age, disease stage, and environmental influences. Therefore, integrating pharmacogenomic approaches with Prakriti assessment may provide a more holistic approach to personalized therapeutics [37].

Drug response may be influenced by Prakriti-related differences in metabolism, drug detoxification, inflammatory status, body composition, gastrointestinal function, and physiological resilience. Drug metabolic capacity and inflammatory responses may differ among Pitta-dominant individuals, who are traditionally associated with higher metabolic and enzymatic activity. Drug distribution patterns may vary among Kapha-dominant individuals because of differences in body composition and metabolism, and among Vata-dominant individuals because of greater sensitivity to neurological stimulation and stress-related effects. These hypotheses require laboratory validation before clinical application [38].

Incorporating Prakriti into pharmacogenomic research may help optimize personalized prescribing, particularly for drugs with narrow therapeutic windows, variable metabolism, or a high potential for adverse effects. It may also support individualized selection of herbal preparations, dosage strategies, and lifestyle-based co-interventions [39]. However, this field remains underdeveloped because of the lack of robust clinical trials correlating Prakriti, pharmacogenomic variants, drug metabolism, and therapeutic efficacy. Further studies are needed to integrate Prakriti assessment with pharmacokinetic profiles, pharmacodynamic endpoints, adverse event monitoring, and genomic drug testing to determine whether constitution-based classification can improve therapeutic precision [40].

Integration of Multi-omics in Ayurgenomic Research

Multi-omics integration involves combining genomics, epigenomics, transcriptomics, proteomics, metabolomics, microbiomics, and clinical phenotyping to provide a systems-level understanding of health and disease. This approach is highly applicable to Ayurgenomics because Prakriti represents a complex phenotype that cannot be explained by a single biomarker or molecular pathway. Instead, Prakriti is likely to reflect interactions among inherited variation, gene regulation, metabolic function, immunity, neuroendocrine function, environmental exposure, and lifestyle adaptation [41].

Using a multi-omics approach, investigators may identify constitution-specific molecular networks and distinguish stable constitutional characteristics from modifiable biological features in the future. Genomics can identify inherited variants; epigenomics can reveal regulatory mechanisms responsive to environmental influences; transcriptomics can characterize active gene expression patterns; proteomics can identify functional protein changes; and metabolomics can capture real-time biochemical states. Along with Prakriti assessment, these datasets may assist in biomarker identification, disease risk prediction, and individualized intervention planning [42].

Artificial intelligence (AI) and bioinformatics could further support Ayurgenomic research by uncovering complex patterns that are difficult to detect using traditional single-variable analyses. Consequently, Prakriti scores, omics data, clinical parameters, lifestyle variables, and disease outcomes can be incorporated into machine learning models to develop predictive tools for personalized healthcare. This approach requires high-quality data, standardized phenotyping, external validation, and clearly defined analytical procedures. Transitioning from small exploratory studies to reproducible multicenter trials will be essential for establishing reliable molecular signatures and integrating Ayurgenomics into evidence-based healthcare. The potential translational framework is summarized in Table 3.

**Table 3:** Epigenomics-Ayurgenomics framework AI: artificial intelligence; DNA: deoxyribonucleic acid; RNA: ribonucleic acid; multi-omics: integrated analysis of multiple molecular data layers.

Translational component	Core approach	Scientific contribution	Clinical or preventive application
Standardized Prakriti assessment	Use of validated questionnaires, expert assessment, and reproducible classification criteria	Improves the reliability of constitution-based phenotyping	Enables consistent patient stratification across clinical and research settings
Epigenomic profiling	Assessment of DNA methylation, histone marks, chromatin state, and non-coding RNAs	Identifies environmentally responsive molecular regulation	Supports lifestyle-sensitive and reversible biomarker discovery
Genomic and pharmacogenomic testing	Analysis of inherited variants and drug-response genes	Clarifies genetic contribution to metabolism, disease risk, and therapeutic response	Supports individualized prescribing and adverse-event reduction
Lifestyle and dietary mapping	Evaluation of diet, sleep, physical activity, stress, seasonality, and environmental exposure	Links Prakriti-specific recommendations with modifiable epigenetic mechanisms	Supports constitution-guided preventive counseling
Multi-omics integration	Combined genomics, epigenomics, transcriptomics, proteomics, metabolomics, and microbiomics	Provides systems-level understanding of Prakriti as a complex phenotype	Enables biomarker panels and predictive health models
AI-assisted prediction	Machine learning models integrating Prakriti scores, molecular data, and clinical variables	Detects complex nonlinear patterns across datasets	Supports risk prediction, clinical decision support, and precision intervention design

Limitations and future directions

This review has several limitations that should be considered when interpreting its findings. The current evidence connecting epigenomics, Ayurgenomics, and Prakriti remains preliminary, with many studies having limited sample sizes, heterogeneous Prakriti assessment methods, and restricted ethnic and geographic representation. Most studies are cross-sectional and, therefore, do not provide information on whether the observed molecular patterns represent stable constitutional markers or are dynamic changes resulting from diet, lifestyle, ageing, environmental influences, or disease status. In addition, the absence of standardized protocols for Prakriti classification, sample collection, omics analysis, and biomarker validation reduces reproducibility and limits direct clinical translation.

Future studies should focus on large-scale, multicenter, longitudinal investigations using validated Prakriti assessment tools and integrated multi-omics platforms. Finally, greater emphasis and funding are needed for epigenomic profiling, pharmacogenomic testing, metabolomic analysis, and the development of AI-based prediction models to identify clinically relevant biomarkers. Prakriti-based dietary, lifestyle, preventive, and therapeutic approaches should be evaluated in well-designed intervention trials to determine their measurable health benefits. Achieving standardized reporting, inclusion of diverse populations, and clinical validation will be crucial for translating Ayurgenomic concepts into evidence-based personalized healthcare.

## Conclusions

This review emphasizes the potential contribution of epigenomics and Ayurgenomics to the molecular understanding of the Ayurvedic concept of Prakriti and its significance in personalized healthcare. Prakriti-based classification represents a model of individual human variability, while epigenomics describes how gene expression may be influenced by diet, lifestyle, stress, environmental factors, and ageing. Integrating these areas could help explain why individuals with different genetic constitutions may have varying susceptibility to disease, metabolic rates, immune responses, stress resilience, and therapeutic responses. Current evidence suggests that genomic, transcriptomic, metabolomic, proteomic, and epigenomic markers may contribute to distinct biological profiles associated with Prakriti. These findings could support early risk prediction, disease prevention, personalized nutrition, lifestyle modification, and pharmacogenomic decision-making. However, several limitations remain in clinical translation, including small sample sizes, non-uniform Prakriti assessment methods, and insufficient biomarker validation. Future research should focus on standardized assessment tools, longitudinal multi-omics studies, and well-designed clinical trials to validate Prakriti-based molecular models. A scientifically rigorous Ayurgenomic approach could support evidence-based, preventive, and individualized healthcare.
